# Cross-Sectional Associations of Reallocating Time Between Sedentary and Active Behaviours on Cardiometabolic Risk Factors in Young People: An International Children’s Accelerometry Database (ICAD) Analysis

**DOI:** 10.1007/s40279-018-0909-1

**Published:** 2018-04-06

**Authors:** Bjørge Herman Hansen, Sigmund Alfred Anderssen, Lars Bo Andersen, Maria Hildebrand, Elin Kolle, Jostein Steene-Johannessen, Susi Kriemler, Angie S. Page, Jardena J. Puder, John J. Reilly, Luis B. Sardinha, Esther M. F. van Sluijs, Niels Wedderkopp, Ulf Ekelund, the International Children’s Accelerometry Database (ICAD) Collaborators, Lars B. Andersen, Sigmund A. Anderssen, Andrew J. Atkin, Rachel Davey, Ulf Ekelund, Dale W. Esliger, Pedro Hallal, Bjørge H. Hansen, Kathleen F. Janz, Susi Kriemler, Niels Møller, Kate Northstone, A. Page, Russel Pate, Jardena J. Puder, John J. Reilly, Jo Salmon, Luis B. Sardinha, Lauren B. Sherar, Anna Timperio, Esther M. F. van Sluijs

**Affiliations:** 1 Department of Sports Medicine, Norwegian School of Sport Sciences, Ullevål Stadion, 0806 Oslo, Norway; 2 Faculty of Teacher Education and Sport, Western Norwegian University of Applied Sciences, Campus Sogndal, Sogndal, Norway; 3 Epidemiology, Biostatistics and Prevention Institute, University of Zürich, Zurich, Switzerland; 4 Centre for Exercise, Nutrition and Health Sciences, University of Bristol, Bristol, UK; 5 Service of Endocrinology, Diabetes and Metabolism, and Division of Pediatric Endocrinology, Diabetes and Obesity, Lausanne University Hospital, Lausanne, Switzerland; 6 Physical Activity for Health Group, School of Psychological Sciences and Health, University of Strathclyde, Glasgow, UK; 7 CIPER, Faculdade de Motricidade Humana, Universidade de Lisboa, Lisbon, Portugal; 8 Centre for Diet and Activity Research (CEDAR) and MRC Epidemiology Unit, University of Cambridge, Cambridge, UK; 9 Department of Regional Health Research, University of Southern Denmark, Odense, Denmark

## Abstract

**Introduction:**

Sedentary time and time spent in various intensity-specific physical activity are co-dependent, and increasing time spent in one behaviour requires decreased time in another.

**Objective:**

The aim of the present study was to examine the theoretical associations with reallocating time between categories of intensities and cardiometabolic risk factors in a large and heterogeneous sample of children and adolescents.

**Methods:**

We analysed pooled data from 13 studies comprising 18,200 children and adolescents aged 4–18 years from the International Children’s Accelerometry Database (ICAD). Waist-mounted accelerometers measured sedentary time, light physical activity (LPA) and moderate-to-vigorous physical activity (MVPA). Cardiometabolic risk factors included waist circumference (WC), systolic blood pressure (SBP), fasting high- and low-density lipoprotein cholesterol (HDL-C and LDL-C), triglycerides, insulin, and glucose. Associations of reallocating time between the various intensity categories with cardiometabolic risk factors were explored using isotemporal substitution modelling.

**Results:**

Replacing 10 min of sedentary time with 10 min of MVPA showed favourable associations with WC, SBP, LDL-C, insulin, triglycerides, and glucose; the greatest magnitude was observed for insulin (reduction of 2–4%), WC (reduction of 0.5–1%), and triglycerides (1–2%). In addition, replacing 10 min of sedentary time with an equal amount of LPA showed beneficial associations with WC, although only in adolescents.

**Conclusions:**

Replacing sedentary time and/or LPA with MVPA in children and adolescents is favourably associated with most markers of cardiometabolic risk. Efforts aimed at replacing sedentary time with active behaviours, particularly those of at least moderate intensity, appear to be an effective strategy to reduce cardiometabolic risk in young people.

## Key Points

**Table:** 

Our results show beneficial theoretical associations between replacing as little as 10 min/day of sedentary time with an equal amount of time spent in moderate-to-vigorous physical activity and a wide array of cardiometabolic risk markers in healthy youth.
Replacing sedentary time with an equal amount of light physical activity showed minor beneficial associations with cardiometabolic risk markers.
Replacing sedentary time with active behaviours, particularly those of at least moderate intensity, appears to be an effective strategy to reduce cardiometabolic risk in young people.

## Introduction

Substituting sedentary time with equal amounts of light physical activity (LPA) or moderate-to-vigorous physical activity (MVPA) and its association with metabolic risk has previously been examined in adults [1]. Generally, these findings indicate that MVPA is the most potent health-enhancing, time-dependent behaviour, while additional benefits are conferred from light-intensity activities when reallocated from sedentary time; however, whether these observations are applicable to children and adolescents is less clear. Replacing time spent sedentary (i.e. 30 min/day) with time spent in MVPA appears favourably associated with markers of adiposity [2, 3]; however, the substitution effects of replacing sedentary time with an equal amount of LPA or MVPA on cardiometabolic risk factors in youth is unknown. Understanding these associations is crucial to inform interventions aimed at reducing sedentary time as well as public health guidelines and policy in children and adolescents, and set the agenda for more precise guidelines for sedentary time in young people that are less well-developed in many countries than physical activity guidelines.

Therefore, the aim of the present study was to examine the theoretical effects of reallocating time between categories of intensities across the intensity spectrum, on markers of cardiometabolic risk factors in a large and heterogeneous sample of children and adolescents in which sedentary time and physical activity were objectively measured in a standardised manner.

## Methods

### Study Design

The International Children’s Accelerometry Database (ICAD; http://www.mrc-epid.cam.ac.uk/research/studies/icad/) is a pooled database containing accelerometer-determined physical activity and sedentary time in children and adolescents from 21 studies from 10 different countries. The aims, design, study selection, inclusion criteria and methods of the ICAD have been described previously [4].

### Participants

For the current analyses, we used data in children and adolescents aged 4–18 years from 13 studies across Australia, Europe, and the US. All included studies provided data on accelerometer-determined sedentary time and physical activity in combination with at least one of the cardiometabolic risk factors of interest. Data collection was performed between 1998 and 2009.

### Assessment of Physical Activity and Sedentary Time

A detailed description of the assessment of physical activity and sedentary time is available elsewhere [4]. Briefly, all raw accelerometer files from all studies included in the ICAD were reprocessed and reanalysed in order to produce physical activity and sedentary time variables that were directly comparable across studies, using specifically developed and commercially available software (KineSoft version 3.3.20, Saskatchewan, Canada; http://www.kinesoft.org). The accelerometer data files from participating studies were reintegrated to 60-s epochs, and non-wear periods were identified (and excluded from further analysis) by scanning the data array for periods of at least 60 min of consecutive zeros (allowing for 2 min of non-zero interruptions). The inclusion criteria for accelerometer data were a valid wear time of ≥ 10 h per day and ≥ 1 day of valid accelerometer data. Individuals with more than 16 h of valid wear time per day were excluded from the analysis as this indicates that the monitor was worn overnight. Intensity thresholds corresponding to sedentary time, LPA and MVPA were derived using the cut-points published by Evenson et al. [5]. Minutes per day spent sedentary and in LPA and MVPA were calculated by summing all minutes where the activity count met the criterion for that intensity, divided by the number of valid days.

### Assessment of Anthropometry and Cardiometabolic Risk Factor Outcomes

Trained personnel measured height and weight by standardised techniques across all studies. We calculated body mass index (BMI) as weight (kilograms) divided by height (metres) squared. For descriptive purposes, we further categorised individuals as normal weight, overweight, and obese based on age- and sex-specific cut-offs [6]. Underweight participants (7% of the participants) were combined with the normal-weight group.

Seven cardiometabolic variables were used as outcomes: abdominal adiposity (waist circumference [WC]) from 13 studies [7–16], resting systolic blood pressure (SBP) from seven studies [7, 9–12, 15, 16], and lipid and glucose metabolism (triglycerides, high-density lipoprotein cholesterol [HDL-C], low-density lipoprotein cholesterol [LDL-C], insulin, and glucose) from seven studies [9–11, 15, 16]. WC was measured with anthropometrical tape around the umbilicus at the end of a normal expiration, except in the National Health and Nutrition Examination Survey (NHANES), where WC was measured just above the iliac crest at the mid-axillary line using similar equipment [16]. All blood pressure measurements were performed in a rested condition using manual or automatic methods and recorded as the average of two, three or four recordings. One study [7] used the DinaMap 9301 vital signs monitor, five studies [9, 10] used the Dinamap XL vital signs monitor, one study [13] used a digital Omron sphygmomanometer and two studies [15, 16] used manual mercury sphygmomanometers. All blood samples were drawn from individuals in a fasted state.

### Statistical Analyses

In order to model the effects of reallocating time between sedentary time, LPA, and MVPA on cardiometabolic risk factors, we used a three-step isotemporal substitution modelling approach. Prior to employing the models, time spent being sedentary or active (LPA and MVPA) was divided by a constant of 10, so that the regression coefficients consistently represent the reallocation of 10 min/day between the various intensity categories. The absolute values of the coefficients will vary if the analyses are performed using different time increments (for example, 1, 30, or 60 min/day) but the direction and relative magnitude of the associations will be identical. First, we examined the association between each of the exposure variables (i.e. time spent sedentary, or in LPA or MVPA) with the individual cardiometabolic risk factors using single-factor models in order to examine the total effect for each behaviour. Second, we used partition models to examine the associations of each exposure variable while controlling for the other exposure variables. We omitted monitor wear time from the partition models because monitor wear time is, by default, the sum of sedentary and active time. Finally, we used isotemporal substitution models to examine the estimated effects of replacing time spent in one behaviour with an equal amount of time spent in another, while keeping monitor wear time constant, by dropping the behaviour of interest from the model. For example, when demonstrating the association between reallocating sedentary time to active time (LPA or MVPA) and the various cardiometabolic risk factors, sedentary time was excluded from the model, while LPA, MVPA, and monitor wear time were kept constant. Using this approach, the regression coefficient for the intensity domains kept in the model (e.g. MVPA) represents the theoretical effect of reallocating an equal amount of time spent in that intensity domain for the same amount of time of the intensity domain excluded (e.g. sedentary time) from the model [17]. The regression coefficients are presented as absolute values and in relative terms.

None of the isotemporal models compromised assumptions of linear regression. The highest observed mean variance inflation factor (VIF) was 1.75, and the lowest tolerance statistic was > 0.62, indicating that multicollinearity was not present.

Participants were recruited from different studies across different countries. We used StataCorp’s xtreg re command (generalised least-square random effects), with study as the panel variable (xtset) and cluster variable (vce cluster option), in all models to obtain robust variance estimations. Due to the large age range in the ICAD database and the known variability in cardiometabolic risk factors by age and pubertal status, individuals were categorised as children (< 10 years), adolescents (10 to < 15 years), and older adolescents (≥ 15 years). In initial analyses, we fitted interaction terms to assess whether sex modified associations between the cardiometabolic risk factors and MVPA within the age categories. We did not observe any significant interactions by sex and therefore combined analysis across sexes, but adjusted for sex. All models were further adjusted for WC, except when it was an outcome variable. The statistical level was set at *p* < 0.05, and was two-sided. All analyses were cross-sectional and were performed using Stata 13.1 (StataCorp. 2013. Stata Statistical Software: TX: StataCorp LP).

## Results

Table 1 presents the descriptive characteristics of the sample stratified by age group. In total, 18,200 individuals with a mean (standard deviation) age of 11.1 years (2.7) [52% females] were included. Based on age- and sex-specific cut-offs for weight status, 25% of the sample was classified as either overweight or obese (20% of the girls and 27% of the boys). Participants wore accelerometers for an average of 13.2 h (1.1) per day, and spent 47% of the time being sedentary, 45% of the time in LPA, and 7% of the time in MVPA.

**Table 1:** Descriptive characteristics of participants by age group [*n* = 18,200]

	Studies^a^	Children	Studies	Adolescents	Studies	Older adolescents
*N*	2–13	4971	1–11	10,836	1, 3–5, 7–8	2393
*N* (%) girls		2573 (51)		5678 (52)		956 (50)
Age, years						
Mean		8.1 (1.8)		11.6 (1.0)		16.2 (0.8)
Range		3.6–9.9		10.0–14.9		15–18.4
Weight, kg		29.5 (8.9)		43.8 (12.4)		65.5 (17.4)
Height, cm		129.5 (12.7)		149.6 (9.2)		168.0 (9.1)
IOTF category						
*N* (%) normal weight		3913 (79)		8092 (75)		1324 (70)
*N* (%) overweight		759 (15)		1991 (18)		356 (19)
*N* (%) obese		295 (6)		734 (7)		216 (11)
Metabolic risk factors						
Waist circumference, cm	2–13	59.1 (8.2)	1–11	68.6 (10.8)	1, 3–5, 7–8	78.1 (14.4)
Systolic blood pressure, mmHg	2–8, 12	99.0 (8.9)	1–8	105.5 (9.8)	3–5, 7–8	110.6 (10.8)
HDL cholesterol, mmol/L	2–8, 11	1.53 (0.32)	2–8, 11	1.43 (0.34)	3–5, 7–8	1.35 (0.30)
LDL cholesterol, mmol/L	2–8, 11	2.57 (0.75)	2–8, 11	2.41 (0.67)	3–5, 7–8	2.39 (0.69)
Insulin, mmol/L	2–5, 11	34.8 (24.7)	2–5, 7–8, 11	66.9 (54.4)	3–5, 7–8	74.3 (64.6)
Triglycerides, mmol/L	2–8, 11	0.74 (0.37)	2–8, 11	0.86 (0.45)	3–5, 7–8	0.90 (0.50)
Glucose, mmol/L	2–5, 11	4.94 (0.52)	2–5, 7–8, 11	5.16 (0.82)	3–5, 7–8	5.14 (0.50)
Physical activity	2–13		1–11		1, 3–5, 7–8	
Wear days		4.0 (1.5)		5.3 (1.7)		4.0 (1.7)
Wear time, min/day		779 (68)		790 (60)		815 (77)
Counts per minute		661 (233)		578 (210)		433 (197)
Sedentary time, min/day		313 (95)		372 (82)		455 (102)
LPA, min/day		405 (72)		361 (65)		318 (83)
MVPA, min/day		56 (31)		54 (29)		39 (29)

Data are expressed as mean (SD) unless otherwise stated

Anthropometric and accelerometer data shown for individuals with at least one metabolic risk factor available. Number of individuals with available data on the various metabolic risk factors are listed in Tables 2–8

*IOTF* International Obesity Task Force, *LPA* light physical activity, *MVPA* moderate-to-vigorous physical activity, *ALSPAC* Avon Longitudinal Study of Parents and Children, *CoSCIS* Copenhagen School Child Intervention Study, *EYHS* European Youth Heart Study, *NHANES* National Health and Nutrition Examination Survey, *PEACH* Personal and Environmental Associations with Children’s Health, *SPEEDY* Sport, Physical Activity and Eating Behavior: Environmental Determinants in Young People, *KISS* Kinder-Sportstudie

^a^Studies: 1: ALSPAC [7]; 2: CoScIS [9]; 3: EYHS Denmark [10]; 4: EYHS Estonia [10]; 5: EYHS Portugal [10]; 6: EYHS Norway [10]; 7: NHANES 03/04 [15]; 8: NHANES 05/06 [16]; 9: PEACH [14]; 10: SPEEDY [12]; 11: KISS [11]; 12: MAGIC [13]; 13: Ballabeina [8]

Reallocating 10 min of daily sedentary time to 10 min of daily MVPA was associated with a reduction in WC across all age groups (Table 2). In children, reallocating 10 min of daily sedentary time to MVPA was associated with a lowering of WC of 0.5% relative to the mean WC in that age group (*b* = − 0.29 cm, 95% confidence interval [CI] − 0.52 to − 0.06). In adolescents and older adolescents, reallocating 10 min of sedentary time to MVPA was associated with a 0.9% (*b* = − 0.59 cm, 95% CI − 0.70 to − 0.48) and 1.0% (*b* = − 0.81 cm, 95% CI − 1.34 to − 0.29) lower WC, respectively. Reallocating sedentary time to LPA was associated with lower WC in adolescents only, but the magnitude of the association was small (< 0.1%).

**Table 2:** Theoretical change in waist circumference (cm) of reallocating 10 min/day between sedentary time, light physical activity and moderate-to-vigorous physical activity among children, adolescent, and older adolescents

	*n*	Sedentary *β* (95% CI)	LPA *β* (95% CI)	MVPA *β* (95% CI)
Children	4971			
Single		0.124 (0.055 to 0.193)**	− 0.069 (− 0.150 to 0.012)	− 0.316 (− 0.571 to − 0.061)*
Partition		0.044 (− 0.002 to 0.091)	− 0.014 (−0.083 to 0.055)	− 0.243 (− 0.473 to − 0.014)*
Isotemporal substitution				
Reduce sedentary		Dropped	− 0.058 (− 0.123 to 0.006)	− 0.287 (− 0.518 to − 0.056)*
Reduce LPA		0.058 (−0.007 to 0.122)	Dropped	− 0.230 (− 0.417 to − 0.044)*
Reduce MVPA		0.290 (0.057 to 0.523)*	0.231 (0.042 to 0.421)*	Dropped
Adolescents	10,836			
Single		0.127 (0.107 to 0.147)**	− 0.097 (− 0.131 to − 0.064)**	− 0.608 (− 0.731 to − 0.484)**
Partition		0.035 (− 0.018 to 0.087)	− 0.012 (− 0.068 to − 0.044)	− 0.555 (− 0.716 to − 0.394)**
Isotemporal substitution				
Reduce sedentary		Dropped	− 0.046 (− 0.073 to − 0.019)*	− 0.589 (−0.702 to − 0.477)**
Reduce LPA		0.047 (0.021 to 0.074)^**^	Dropped	− 0.541 (− 0.659 to − 0.424)**
Reduce MVPA		0.601 (0.491 to 0.712)**	0.559 (0.444 to 0.674)**	Dropped
Older adolescents	1896			
Single		− 0.007 (− 0.049 to 0.035)	− 0.038 (− 0.106 to 0.031)	− 0.406 (− 0.874 to − 0.063)*
Partition		− 0.125 (− 0.255 to − 0.006)	− 0.153 (− 0.28 to −0.028)*	− 0.939 (− 1.436 to − 0.441)**
Isotemporal substitution				
Reduce sedentary		Dropped	− 0.027 (− 0.131 to 0.078)	− 0.814 (− 1.334 to −0.294)*
Reduce LPA		0.029 (− 0.076 to 0.134)	Dropped	− 0.784 (− 1.233 to − 0.336)*
Reduce MVPA		0.834 (0.304–1.364)*	0.813 (0.352–1.275)*	Dropped

*LPA* light physical activity, *MVPA* moderate-to-vigorous physical activity, *CI* confidence interval

*Significant at *p* < 0.05, **significant at *p* ≤ 0.001

Reallocating sedentary time to either LPA or MVPA was associated with lower SBP among adolescents, but not in children and older adolescents (Table 3). Reallocating 10 min of daily sedentary time to MVPA was associated with an absolute lowering of SBP of 0.08 mmHg (95% CI − 0.14 to − 0.03) in the adolescent group, equal to a relative lowering of SBP of 0.1%.

**Table 3:** Theoretical change in systolic blood pressure (mmHg) of reallocating 10 min/day between sedentary time, light physical activity and moderate-to-vigorous physical activity among children, adolescents and older adolescents

	*n*	Sedentary *β* (95% CI)	Light PA *β* (95% CI)	MVPA *β* (95% CI)
Children	3544			
Single		− 0.008 (− 0.034 to 0.018)	− 0.006 (− 0.041 to 0.030)	− 0.091 (− 0.237 to 0.055)
Partition		− 0.054 (− 0.098 to − 0.010)**	− 0.027 (− 0.092 to 0.037)	− 0.151 (− 0.320 to 0.019)
Isotemporal substitution				
Reduce sedentary		Dropped	0.027 (− 0.005 to 0.058)	− 0.097 (− 0.250 to 0.055)
Reduce light PA		− 0.027 (− 0.059 to 0.005)	Dropped	− 0.124 (− 0.294 to 0.045)
Reduce MVPA		0.096 (− 0.057 to 0.248)	0.122 (− 0.047 to 0.292)	Dropped
Adolescents	8100			
Single		0.032 (0.003 to 0.061)*	− 0.039 (− 0.060 to − 0.018)**	− 0.107 (− 0.179 to −0.035)*
Partition		0.005 (− 0.033 to 0.044)	− 0.025 (− 0.050 to − 0.000)*	− 0.079 (− 0.122 to − 0.036)*
Isotemporal substitution				
Reduce sedentary		Dropped	− 0.031 (− 0.051 to − 0.010)*	− 0.084 (− 0.143 to − 0.025)*
Reduce light PA		0.030 (0.010–0.050)*	Dropped	− 0.056 (− 0.010 to − 0.012)*
Reduce MVPA		0.083 (0.026 to 0.139)*	0.052 (0.011 to 0.092)*	Dropped
Older adolescents	1865			
Single		0.046 (− 0.009 to 0.102)	− 0.036 (− 0.101 to 0.029)	− 0.217 (− 0.507 to 0.072)
Partition		0.058 (− 0.022 to 0.138)	0.019 (− 0.055 to 0.094)	− 0.184 (− 0.408 to 0.041)
Isotemporal substitution				
Reduce sedentary		Dropped	− 0.039 (− 0.120 to 0.042)	− 0.240 (− 0.535 to 0.053)
Reduce light PA		0.039 (− 0.041 to 0.119)	Dropped	− 0.202 (− 0.467 to 0.062)
Reduce MVPA		0.243 (− 0.048 to 0.535)	0.206 (−0.055 to 0.467)	Dropped

*LPA* light physical activity, *MVPA* moderate-to-vigorous physical activity, *CI* confidence interval

*Significant at *p* < 0.05, **significant at *p* ≤ 0.001

Reallocating time from sedentary to active time was not associated with HDL-C, however replacing LPA with MVPA yielded a small but significantly relative higher HDL-C of 0.7% (*b* = 0.01 mmol/L, 95% CI 0.00–0.02) in the youngest age group (Table 4). Reallocating time between sedentary and active time was favourably associated with LDL-C among children and adolescents, showing a 0.9% (*b* = − 0.02 mmol/L, 95% CI − 0.03 to − 0.01) and 0.5% (*b* = − 0.01 mmol/L, 95% CI − 0.03 to − 0.00) lower LDL-C, respectively, when replacing sedentary time with MVPA (Table 5). Reallocating sedentary time to LPA unexpectedly showed a small, undesirable positive association with LDL-C in the two youngest age groups. Reallocating 10 min of daily sedentary time to MVPA was favourably associated with 2.2% lower triglycerides (*b* = − 0.02 mmol/L, 95% CI − 0.03 to − 0.01) in the adolescent group (Table 6).

**Table 4:** Theoretical change in high-density lipoprotein cholesterol (mmol/L) of reallocating 10 min/day between sedentary time, light physical activity, and moderate-to-vigorous physical activity among children, adolescents and older adolescents

	*n*	Sedentary *β* (95% CI)	Light PA *β* (95% CI)	MVPA *β* (95% CI)
Children	3112			
Single		0.001 (− 0.002 to 0.004)	− 0.000 (− 0.003 to 0.003)	0.006 (− 0.002 to 0.014)
Partition		0.003 (0.000 to 0.005)*	− 0.001 (− 0.004 to 0.002)	0.009 (− 0.001 to 0.014)
Isotemporal substitution				
Reduce sedentary		Dropped	− 0.004 (− 0.008 to 0.000)	0.006 (− 0.001 to 0.013)
Reduce light PA		0.004 (0.000 to 0.008)	Dropped	0.010 (0.001 to 0.019)*
Reduce MVPA		− 0.006 (− 0.014 to 0.002)	− 0.010 (− 0.019 to − 0.002)*	Dropped
Adolescents	2135			
Single		− 0.001 (− 0.003 to − 0.000)*	0.000 (− 0.001 to 0.002)	0.007 (− 0.002 to 0.017)
Partition		− 0.002 (− 0.002 to − 0.001)**	− 0.002 (− 0.004 to −0.001)*	0.005 (− 0.006 to 0.015)
Isotemporal substitution				
Reduce sedentary		Dropped	− 0.000(− 0.002 to 0.001)	0.006 (− 0.005 to 0.018)
Reduce light PA		0.000 (− 0.001 to 0.002)	Dropped	0.006 (− 0.006 to 0.019)
Reduce MVPA		− 0.006 (− 0.018 to 0.005)	− 0.007 (− 0.019 to 0.006)	Dropped
Older adolescents	1788			
Single		− 0.000 (− 0.002 to 0.001)	0.001 (0.000 to 0.002)*	0.002 (− 0.002 to 0.006)
Partition		0.001 (− 0.000 to 0.002)	0.001 (− 0.001 to 0.003)	0.002 (− 0.001 to 0.005)
Isotemporal substitution				
Reduce sedentary		Dropped	0.000 (− 0.003 to 0.003)	0.000 (− 0.004 to 0.003)
Reduce light PA		− 0.001 (− 0.002 to 0.001)	Dropped	0.001 (− 0.005 to 0.002)
Reduce MVPA		− 0.000 (− 0.003 to 0.004)	− 0.001 (− 0.002 to 0.004)	Dropped

*LPA* light physical activity, *MVPA* moderate-to-vigorous physical activity, *CI* confidence interval

*Significant at *p* < 0.05, **significant at *p *≤ 0.001

**Table 5:** Theoretical change in low-density lipoprotein cholesterol (mmol/L) of reallocating 10 min/day between sedentary time, light physical activity and moderate-to-vigorous physical activity among children, adolescents and older adolescents

	*n*	Sedentary *β* (95% CI)	Light PA *β* (95% CI)	MVPA *β* (95% CI)
Children	2428			
Single		− 0.002 (− 0.002 to 0.006)	0.000 (− 0.002 to 0.004	− 0.020 (− 0.030 to − 0.010)**
Partition		− 0.002 (− 0.007 to 0.003)	0.002 (− 0.002 to 0.005)	− 0.024 (− 0.032 to − 0.016)**
Isotemporal substitution				
Reduce sedentary		Dropped	0.003 (0.001–0.006)*	− 0.022 (− 0.032 to − 0.012)**
Reduce light PA		− 0.004 (− 0.006 to −0.001)*	Dropped	− 0.026 (− 0.035 to − 0.016)**
Reduce MVPA		0.022 (0.012 to 0.032)**	0.026 (0.016 to 0.035)**	Dropped
Adolescents	1166			
Single		− 0.003 (− 0.006 to 0.001)	0.004 (− 0.001 to 0.010)	− 0.007 (− 0.021 to 0.007)
Partition		− 0.004 (− 0.009 to 0.001)	0.003 (− 0.003 to 0.009)	− 0.016 (− 0.030 to − 0.001)*
Isotemporal substitution				
Reduce sedentary		Dropped	0.003 (0.001 to 0.011)*	− 0.012 (− 0.023 to 0.001)*
Reduce light PA		− 0.006 (− 0.011 to − 0.001)^*^	Dropped	− 0.018 (− 0.029 to − 0.007)*
Reduce MVPA		0.012 (0.001 to 0.024)	0.018 (0.007–0.029)	Dropped
Older adolescents	1222			
Single		0.003 (0.001 to 0.006)*	− 0.005 (− 0.010 to 0.000)*	− 0.023 (− 0.034 to − 0.012)**
Partition		− 0.000 (− 0.005 to 0.005)	− 0.001 (− 0.005 to 0.003)	0.003 (− 0.018 to 0.025)
Isotemporal substitution				
Reduce sedentary		Dropped	− 0.001 (− 0.005 to 0.003)	0.003 (− 0.022 to 0.029)
Reduce light PA		− 0.001 (− 0.003 to 0.005)	Dropped	0.004 (− 0.019 to 0.027)
Reduce MVPA		− 0.005 (− 0.031 to 0.022)	− 0.006 (− 0.030 to 0.018)	Dropped

*LPA* light physical activity, *MVPA* moderate-to-vigorous physical activity, *CI* confidence interval

*Significant at *p* < 0.05, **significant at *p* ≤ 0.001

**Table 6:** Theoretical change in triglycerides (mmol/L) of reallocating 10 min/day between sedentary time, light physical activity and moderate-to-vigorous physical activity among children, adolescents and older adolescents

	*n*	Sedentary *β* (95% CI)	Light PA *β* (95% CI)	MVPA *β* (95% CI)
Children	2415			
Single		0.002 (− 0.001 to 0.004)	− 0.003 (− 0.005 to 0.001)	− 0.008 (− 0.015 to − 0.000)*
Partition		− 0.001 (− 0.005 to 0.003)	− 0.002 (− 0.004 to 0.001)	− 0.007 (− 0.016 to − 0.003)*
Isotemporal substitution				
Reduce sedentary		Dropped	− 0.001 (− 0.003 to 0.001)	− 0.006 (− 0.013 to 0.001)
Reduce light PA		0.001 (− 0.001 to 0.003)	Dropped	− 0.005 (− 0.013 to 0.003)
Reduce MVPA		0.006 (− 0.001 to 0.013)	0.005 (− 0.003 to 0.013)	Dropped
Adolescents	1160			
Single		0.001 (− 0.003 to 0.004)	− 0.001 (− 0.005 to 0.004)	− 0.017 (− 0.022 to − 0.011)*
Partition		− 0.003 (− 0.006 to 0.001)	0.000 (− 0.003 to 0.002)	− 0.021 (− 0.030 to − 0.013)**
Isotemporal substitution				
Reduce sedentary		Dropped	0.002 (− 0.003 to 0.008)	− 0.019 (− 0.026 to − 0.012)**
Reduce light PA		− 0.002 (− 0.008 to 0.003)	Dropped	− 0.021 (− 0.032 to − 0.010)**
Reduce MVPA		0.019 (0.012 to 0.026)**	0.021 (0.010 to 0.032)**	Dropped
Older adolescents	1224			
Single		0.000 (− 0.003 to 0.003)	− 0.004 (− 0.008 to − 0.001)**	− 0.005 (− 0.019 to − 0.010)*
Partition		− 0.004 (− 0.011 to 0.004)	− 0.008 (− 0.014 to − 0.002)*	− 0.011 (− 0.020 to − 0.002)*
Isotemporal substitution				
Reduce sedentary		Dropped	− 0.004 (− 0.007 to − 0.001)*	− 0.007 (− 0.014 to 0.001)
Reduce light PA		0.004 (0.001 to 0.007)*	Dropped	− 0.003 (− 0.012 to 0.006)
Reduce MVPA		0.006 (− 0.002 to 0.014)	0.002 (− 0.008 to 0.011)	Dropped

*LPA* light physical activity, *MVPA* moderate-to-vigorous physical activity, *CI* confidence interval

*Significant at *p* < 0.05, **significant at *p *≤ 0.001

Reallocating sedentary time to LPA or MVPA was favourably associated with insulin in the youngest age group. In this group, reallocating 10 min of daily sedentary time to MVPA was associated with a 2.4% (*b* = − 0.85 mmol/L, 95% CI − 1.61 to − 0.09) lower fasting insulin (Table 7). The variable insulin was skewed, but analyses of the log transformed values showed similar patterns, with the only meaningful exception being that the nonsignificant negative association of reallocating sedentary time to MVPA for the adolescents group became significant. Lastly, reallocating sedentary time to MVPA was favourably associated with glucose in the two oldest age groups (Table 8). Reallocating 10 min of sedentary time to MVPA was associated with a 0.4% lower fasting glucose for adolescents (*b* = − 0.02 mmol/L, 95% CI − 0.03 to − 0.00) and older adolescents (*b* = − 0.02 mmol/L, 95% CI − 0.03 to − 0.00). In the oldest age group, reallocating sedentary time to LPA was also favourably associated with glucose, but the order of magnitude was small (< 0.1%).

**Table 7:** Theoretical change in insulin (mmol/L) of reallocating 10 min/day between sedentary time, light physical activity and moderate-to-vigorous physical activity among children, adolescents and older adolescents

	*n*	Sedentary *β* (95% CI)	Light PA *β* (95% CI)	MVPA *β* (95% CI)
Children	2151			
Single		− 0.127 (− 0.393 to 0.140)	0.073 (− 0.092 to 0.238)	− 0.677 (− 1.301 to −0.052)*
Partition		− 0.151 (− 0.488 to 0.186)	0.296 (0.081 to 0.510)*	− 1.000 (− 1.976 to − 0.023)*
Isotemporal substitution				
Reduce sedentary		Dropped	0.446 (0.107 to 0.785)*	− 0.850 (− 1.614 to − 0.086)*
Reduce light PA		− 0.448 (− 0.785 to − 0.111)*	Dropped	− 1.295 (− 2.171 to − 0.419)*
Reduce MVPA		0.847 (0.093 to 1.600)*	1.291 (0.426 to 2.155)*	Dropped
Adolescents	1049			
Single		0.136 (− 0.003 to 0.275)	0.030 (− 0.162 to 0.221)	− 0.659 (− 1.607 to 0.290)
Partition		0.226 (− 0.070 to 0.521)	0.322 (− 0.062 to 0.707)	− 0.547 (− 1.558 to 0.464)
Isotemporal substitution				
Reduce sedentary		Dropped	0.094 (− 0.025 to 0.213)	− 0.770 (− 1.745 to 0.205)
Reduce light PA		− 0.101 (− 0.215 to 0.012)	Dropped	− 0.877 (− 1.897 to 0.144)
Reduce MVPA		0.727 (− 0.293 to 1.474)	0.817 (− 0.271 to 1.905)	Dropped
Older adolescents	1222			
Single		− 0.206 (− 0.51 to 0.107)	− 0.067 (− 0.313 to 0.179)	− 0.255 (− 1.474 to 0.964)
Partition		− 0.371 (− 0.684 to − 0.058)*	− 0.355 (− 0.540 to − 0.171)**	− 0.276 (− 1.822 to 1.269)
Isotemporal substitution				
Reduce sedentary		Dropped	0.017 (− 0.411 to 0.446)	− 0.092 (− 1.633 to 1.818)
Reduce light PA		− 0.020 (− 0.450 to 0.408)	Dropped	0.068 (− 1.468 to 1.604)
Reduce MVPA		0.123 (− 0.870 to 1.625)	0.109 (− 1.674 to 1.456)	Dropped

*LPA* light physical activity, *MVPA* moderate-to-vigorous physical activity, *CI* confidence interval

*Significant at *p *< 0.05, **significant at *p *≤ 0.001

**Table 8:** Theoretical change in glucose (mmol/L) of reallocating 10 min/day between sedentary time, light physical activity and moderate-to-vigorous physical activity among children, adolescents and older adolescents

	*n*	Sedentary *β* (95% CI)	Light PA *β* (95% CI)	MVPA *β* (95% CI)
Children				
Single	2165	0.001 (− 0.001 to 0.003)	− 0.003 (− 0.005 to − 0.000)	− 0.004 (− 0.013 to 0.005)
Partition		0.011 (− 0.008 to 0.030)	0.001 (− 0.013 to 0.015)	0.006 (− 0.010 to 0.021)
Isotemporal substitution				
Reduce sedentary		Dropped	−0.010 (− 0.021 to − 0.000)*	− 0.006 (− 0.018 to 0.007)
Reduce light PA		0.010 (0.000 to 0.021)*	Dropped	0.005 (− 0.011 to 0.020)
Reduce MVPA		0.005 (− 0.007 to 0.017)	− 0.005 (− 0.021 to 0.011)	Dropped
Adolescents				
Single	1060	0.000 (− 0.002 to 0.003)	− 0.006 (− 0.014 to 0.003)	− 0.018 (− 0.035 to − 0.002)*
Partition		− 0.012 (− 0.023 to 0.000)	− 0.013 (− 0.030 to 0.003)	− 0.027 (− 0.050 to −0.004)*
Isotemporal substitution				
Reduce sedentary		Dropped	− 0.002 (− 0.006 to 0.003)	− 0.016 (− 0.030 to − 0.002)*
Reduce light PA		0.001 (− 0.003 to 0.006)	Dropped	− 0.013 (−0.024 to − 0.002)*
Reduce MVPA		0.015 (0.001 to 0.030)*	0.014 (0.000 to 0.027)*	Dropped
Older adolescents				
Single	1232	− 0.002 (− 0.006 to 0.003)	0.002 (− 0.001 to 0.006)	− 0.015 (− 0.028 to − 0.002)*
Partition		− 0.003 (− 0.008 to 0.003)	− 0.002 (− 0.002 to 0.006)	− 0.017 (− 0.028 to − 0.006)*
Isotemporal substitution				
Reduce sedentary		Dropped	0.005 (0.001–0.008)*	− 0.015 (− 0.026 to − 0.003)*
Reduce light PA		− 0.005 (− 0.008 to − 0.001)*	Dropped	− 0.019 (− 0.028 to − 0.010)**
Reduce MVPA		0.015 (0.003 to 0.026)*	0.019 (0.009 to 0.029)**	Dropped

*LPA* light physical activity, *MVPA* moderate-to-vigorous physical activity, *CI* confidence interval

*Significant at *p *< 0.05, **significant at *p *≤ 0.001

## Discussion

We examined the theoretical effects of reallocating time between sedentary and active behaviours on seven commonly reported cardiometabolic risk factors in children and adolescents. Our results suggest, with some exceptions, that the intensity of activity required for health benefits needs to be of at least moderate intensity to influence cardiometabolic risk factors in children and adolescents. These findings support the current physical recommendations focusing on the health-enhancing effects of at least moderate physical activity.

The magnitude of associations when examining the effect of reallocating sedentary time to MVPA on WC corroborates previous observations in comparable age groups. Studies have shown that reallocating 30 min of sedentary time per day to MVPA was associated with a 1.3 cm reduction in WC in a sample of Portuguese children [3], and reallocating 60 min/day of sedentary time to MVPA was associated with a 3.8 cm lower WC in a sample of American children [18]. In comparison, the observed lowering of WC would be 3.0 cm if we examined the reallocation of 60 min of MVPA instead of 10 min. In the present study, we show the beneficial associations of replacing 10 min of sedentary time with MVPA in order to illustrate the beneficial association of a feasible reallocation of time. Reallocating 60 min of sedentary time to MVPA would, for the 9-year-olds in the included sample, mean an average relative increase in MVPA by 111%, which might not be possible. The more feasible reallocation of 10 min is of relevance to public health as childhood obesity is a risk factor for atherosclerosis and is associated with increased mortality due to cardiovascular disease in adulthood [19]. Furthermore, childhood obesity is associated with a clustering of risk factors, such as elevated blood pressure, triglycerides, (reduced) HDL-C and LDL-C, and insulin [20], independent of the definition used for obesity and the methods used for central body fat deposition in children and adolescents [21].

The present study showed beneficial, albeit small, associations with reallocating sedentary time to MVPA on SBP, independent of WC, among adolescents. The primary and secondary preventive effects of physical activity on both normo- and hypertensive adults are well-established, but are less clear in children and adolescents. Although the magnitude of associations were small, our observation is novel as it has been suggested that physical activity may not affect BP values in normotensive adolescents [22]. We speculate that the lack of beneficial associations for BP in the youngest and oldest age groups, as well as the modest magnitude of associations, might be attributed to not including vigorous intensity physical activity as a separate component of the physical activity intensity spectrum. Furthermore, the potential underestimation of vigorous physical activity when using 60-s epochs in paediatric samples might further attenuate the associations as studies have indicated that interventions aimed at reducing SBP in normotensive adolescents should involve vigorous physical activity [23].

The included risk factors reflecting lipid (HDL-C, LDL-C, and triglycerides) and carbohydrate metabolism (glucose and fasting insulin) showed similar patterns of associations with the exposures. Although the associations were significant, relative sizes (0.1–1.0%) were small and must be interpreted with caution. Observational studies in young people most commonly show weak associations between physical activity and markers of lipid metabolism [24]; however, beneficial associations are usually observed in intervention studies where interventions proved effective in increasing aerobic fitness [25, 26]. For some of the cardiometabolic risk factors (insulin in the youngest age group; LDL-C and glucose among the oldest age group), reallocating sedentary time into LPA was associated with a worsening of the cardiometabolic risk factor. Although these associations were weak (< 0.1%), and thus of less clinical importance, they were unexpected and might not represent true effects. They might be chance findings or more likely due to misclassification of LPA and sedentary time [27, 28]. Both Kwon et al. [29] and Carson et al. [30] showed consistently stronger associations between LPA and adiposity using higher versus lower cut-points for LPA, indicating that the cut-point used in our study for LPA might not adequately differentiate between sedentary time and LPA.

Several limitations and strengths warrant mentioning when interpreting the findings of our study. The cross-sectional design of the analyses limits establishing causal relationships. However, with the exception for WC, it is unlikely that metabolic risk factors lead to lower levels of physical activity, whereas it is biologically plausible that LPA, and specifically MVPA, affect the metabolic risk factors. Indeed, previous studies have suggested an inverse association between MVPA and cardiometabolic risk factors independent of sedentary time, adiposity and other confounders [31]. Furthermore, well-known limitations associated with accelerometers may affect the results. These include the use of 60-s epoch periods, which may underestimate MVPA in children and adolescents, and the fact that the hip-worn accelerometers do not differentiate between standing and sitting, which are two inherently different behaviours. Furthermore, activities with little or no vertical movement of the hip (e.g. bicycling), as well as water-based activities (when the monitor was removed), are likely to be misclassified. Although random measurement error attenuates, rather than biases, observed associations, we cannot rule out regression dilution bias due to unmeasured activities such as bicycling (e.g. frequent cyclers having lower accelerometer-determined PA levels but also a healthier cardiometabolic risk factor profile [32]) or swimming (monitor removed for water activities). We also acknowledge that unmeasured confounders such as diet, race, pubertal status and socioeconomic position may have introduced bias, increasing or decreasing the ability of the current analyses to determine significant associations. Furthermore, the large sample size included in the current study might lead to small and significant associations that may be less clinically important. However, activity levels of children are highly variable and our estimates of the childrens’ activity levels may not be fully representative, likely underestimating the ‘true’ association with risk markers observed when reallocating 10 min between the various indices of intensity. Indeed, the within-individual intraclass correlation coefficient for objectively measured physical activity in children has been estimated to be 0.5, indicating that the ‘true’ magnitude of association between physical activity and cardiometabolic outcomes might be at least twice as strong as observed in the present study [33].

Finally, these associations were observed in generally healthy children and adolescents where cardiometabolic risk factors are less well-established, and single risk factors may relate weakly to physical activity intensity due to day-to-day fluctuations in both risk factors and patterns of physical activity [10]. Despite this, the pathogenesis of atherosclerosis may begin at an early age [34], and individual cardiometabolic risk factors are shown to track from childhood to adulthood [35–37]. Thus, weak associations observed in childhood may transfer into substantial health problems later in life, especially when considered at the level of the population.

The strengths of the study include the large and heterogeneous sample of young people in which cardiometabolic risk factors were available in combination with objectively measured sedentary time and physical activity. All individual accelerometer data files were cleaned, processed and reanalysed in a standardised manner, allowing pooling of data from several studies. Lastly, the isotemporal substitution modelling framework is a novel analytical method to examine the theoretical effects of displacing a fixed duration of time between the various intensity domains.

## Conclusions

Efforts aimed at replacing sedentary time with active behaviours could be effective in relation to a wide array of cardiometabolic risk factors in young people. Although some favourable associations were observed when replacing sedentary time with LPA, stronger and more consistent associations were observed when substituting sedentary time with MVPA, for most of the risk factors across age groups. Replacing LPA with MVPA showed similar effect sizes as replacing sedentary time with MVPA. These findings have important implications for public health and suggest public health efforts should continue to strive to increase physical activity of at least moderate intensity in youth.
